# Supplementary material for: Are carbon emissions trading and green financial instruments synergistic? -Comprehensive quantitative research based on content analysis
Source: PLoS One. 2024 Mar 7;19(3):e0298601. doi: 10.1371/journal.pone.0298601 (PMC10919601; doi:10.1371/journal.pone.0298601)
Supplement: S1 Data — (ZIP) [file pone.0298601.s001.zip › Data/地级市Z_F工作报告.zip!/╔╜╬≈/╘╦│╟╩╨/└┤╫╘╣1⁄2╓┌║┼í╛┬φ┐╦╩2╛▌═°í┐_2019.docx]

各位代表：

现在，我代表市人民政府向大会报告工作，请予审议，并请市政协委员和其他列席人员提出意见。

一、2018年工作回顾

2018年是运城发展历程上不平凡的一年。面对经济下行压力加大的严峻形势，在市委的坚强领导下，全市上下坚持以习近平新时代中国特色社会主义思想为指导，认真贯彻党的十九大精神和习近平总书记视察山西重要讲话精神，按照省委“一个指引、两手硬”工作思路和要求，紧盯“示范区”“排头兵”“新高地”三大目标，以“改革抢先机，发展站前列，各项工作创一流”为总要求，以“走进新时代，建设大运城”为总抓手，坚持稳中求进工作总基调，全力以赴稳增长、促改革、调结构、惠民生、防风险，实现了经济增长保持中高速、全省排位保持中上游的目标。全市地区生产总值增长7%，规模以上工业增加值增长7%，社会消费品零售总额增长8.3%，一般公共预算收入增长20.3%，城乡居民人均可支配收入分别增长6.6%和9.2%，居民消费价格涨幅1.5%，全市转型发展、高质量发展取得新成效。

这一年，我们倾力推进转型发展，进一步向实体经济聚焦发力，新型工业化迈出坚实步伐。“三个发展计划”支撑有力。“龙腾虎跃”计划带动骨干企业强势崛起，阳光焦化、高义钢铁荣登“龙榜”，“龙榜”企业累计达到4户，山西建龙首家突破200亿元；“虎榜”企业净增9户，达到34户，“龙虎榜”培育企业总产值占全市工业的79.7%。“群星灿烂”计划引领小微企业发展壮大，成功举办首届工博会，新认定国家级“双创”基地1家、省级5家、市级14家，培育“小升规”企业60户，孵化创办小微企业8892户，创造就业岗位8.4万个。“凤还巢”计划展现旺盛活力，建立了市县乡村四级在外务工人员服务体系和大数据管理平台，7362名本土优秀人才回乡创业，创办规模企业133户、小微企业1152户，吸引各类投资139亿元。项目建设卓有成效。实施“3515重大工程项目”“龙腾虎跃千亿工程”“城建111工程”等系列重点项目，运城国际机场扩建、动车存车场扩建等项目集中开工，运宝黄河大桥即将通车，建龙烧结机配套、中铝新材料氧化铝等项目如期投产，全市转型项目投资占比达到65.4%。工业技术改造力度加大。实施传统产业技术改造三年专项行动，全市技改投资同比增长31.6%，大运高端牵引车、中条山有色制备陶瓷、同誉轮毂等36个项目获得2.1亿元的省技改转型资金支持。装备制造、新材料、新能源等新兴产业全面发展，非煤产业占比达到86%，位列全省第一。“三大基地”建设扎实推进。编制了国家新型铝镁合金产业基地实施方案和中西部精品钢基地、汽车及新能源汽车基地发展规划，谋划实施总投资252.2亿元的27个重大项目，“三大基地”发展呈现良好态势。创新驱动能力明显增强。新认定6家省级企业技术中心、14家市级企业技术中心。4家企业取得国家级“两化”融合管理体系评定证书，11家企业分别获批国家级、省级“两化”融合管理体系贯标试点。高新技术企业总数突破120家，发明专利拥有量达到826件。省级众创空间和科技企业孵化器达到9家。科技大市场年技术合同登记交易额达到23.4亿元，同比增长48%。与中兴合作建设的大数据中心即将投入运营。

这一年，我们大力实施乡村振兴战略，持续推进“八大提档升级行动”，农业农村现代化进程全面加快。农业生产再获丰收。全年粮食总产28.7亿公斤，超省定任务14.6%；蔬菜产量20.9亿公斤，增长5.2%；肉类总产量2.2亿公斤，增长17.7%。农业基础设施不断加强。建设高标准农田48.6万亩；新建末级渠系配套工程330.8公里，新增高效节水灌溉面积13万亩，全市农业实际灌溉面积达到700万亩，基本实现主要农田黄河引水灌溉全覆盖。主要农作物耕种收综合机械化率达到73.2%。农产品出口平台建设初见成效。持续推进优质农产品出口生产、检验检疫和贸易营销“三大体系”建设，农产品出口服务中心正式运行，认证出口果园195个，辐射带动110余万亩优质果品基地，万荣苹果产业园获批全省唯一国家级现代农业产业园，稷山省级农产品出口质量安全示范区成功创建。万荣、盐湖入选首批国家农村产业融合发展示范园。成功举办第三届果博会，编制并发布全国首个苹果主产区现货价格指数——“新华—中国（运城）苹果价格指数”，农产品出口3.8亿公斤，出口国家和地区新增5个、达到59个。农村改革稳步开展。99.8%的行政村完成了确权登记工作，98.3%的确权农户领到新版承包经营权证，各县（市、区）均建成了农村产权流转交易市场，全市土地流转面积221.1万亩，占家庭承包经营耕地总面积的28.1%。农村集体产权制度改革稳步推进，全国试点河津市和省级试点临猗县完成清产核资和成员身份认定。农村人居环境明显改善。全面深化“四治六化一创”农村环境集中整治，建成348个“美丽宜居示范村”。芮城县被授予“国家生态文明建设示范县”称号。

这一年，我们强化规划引领，着力推进“一区五带”发展布局，城乡一体化步伐加快。制定《运城市“一区五带”产业发展规划》，有序推进优势产业和特色产业向不同主体功能区聚集；“盐临夏”一体化深入推进，夏县撤县设区工作已上报国务院待批。启动2035版城市总体规划修编、东部片区、北部新城片区和官道河水系等部分专项规划，推进了“多规合一”规划及大数据平台建设，强化了空间管控能力。中心城区人民北路畅通、学苑路提升改造工程竣工通车，魏风街、大禹街等道路相继竣工，热电联产集中供热二期项目投入运行。体育公园建成开放，市城区190条小街小巷改造任务全面完成。全市建成64座乡（镇）垃圾中转站，新开工棚户区改造住房7160套，基本建成棚户区改造安置房、公共租赁住房9629套，建成率为省定任务的148.1%。智慧运城建设成效显著，“智慧城市云平台”及教育、食药、政务、医疗、城管五大应用投入使用，其中智慧教育被认定为全国试点平台。

这一年，我们力促融合融通，培育壮大支柱产业，现代服务业发展势头强劲。文化旅游业快速发展。按照全省打造黄河旅游板块的战略部署，扎实推进总投资81亿元的沿黄12个重点旅游项目。成功举办“三节三会”等一系列主题活动，召开首届旅游发展大会，与中青旅、东方文旅等公司签署战略合作框架协议，推动全域旅游发展。永济市喜获央视“十佳魅力城市”称号。全市年接待游客人次和旅游总收入增幅分别达24.6%和26.9%。金融支持实体经济力度加大。金融业实现增加值89.9亿元，增长5.2%；金融支持“三个发展计划”信贷投放432亿元，处置不良贷款59.1亿元。新增“新三板”挂牌企业2家，累计达15家。成立了总规模67亿元的7只基金，正在加紧设立转型发展基金、文旅发展基金、农业发展基金等总规模222亿元的6只基金。商贸物流业快速发展。全市形成星河电商产业园等6个电商集聚区，在淘宝、京东等电商平台开立网店1.1万余家。万达广场等大型商业综合体相继开业运营。

这一年，我们全面深化改革，努力扩大对外开放，全市经济发展的动力和活力显著增强。医药卫生体制改革走在全国前列。全国、全省县域综合医改现场会先后在我市召开，盐湖区医疗机构一体化改革受到国务院表扬。开发区改革持续深化。稷山、新绛、垣曲开发区获批，全市省级开发区达到10个，位列全省第一。深入推进开发区“三化三制”改革，企业项目投资承诺制全面实行。国资国企改革深入推进。实施僵尸企业3年出清计划，清华水泥厂等5家企业进入改制程序。全面完成国有企业“三供一业”分离移交工作，涉及7万余户。对外开放水平全面提升。山西自由贸易试验区运城片区申报工作进展顺利。运城机场年旅客吞吐量突破200万人次，航空口岸正式开放报国务院待批。高铁客运量超过900万人次，增长45%。与41个“一带一路”国家建立了贸易往来关系，进出口实绩企业达到146家。

这一年，我们践行绿色发展理念，强力推进环保治理，生态文明建设取得突破性进展。环保督察问题整改扎实有效。中央、省环保督察反馈问题全面达成整改要求。“三大保卫战”成效明显。大力推进电力、钢铁、焦化等行业提标改造，新增集中供热面积1465.6万平方米，完成“煤改气”“煤改电”5万余户，中心城区基本实现清洁取暖全覆盖，环境空气质量综合指数同比下降14.5%，优良天气达标天数增加15天；取缔“散乱污”企业3976家；成功申报国家冬季清洁取暖试点城市，获得3年9亿元的资金支持。建成14个沿汾河农村生活污水处理站，完成4个国考断面水质自动监测站建设，汾河、涑水河流域生态修复工程扎实推进；省考核11个水质断面中，优良断面占54.5%，比考核目标高18个百分点。开展农用地土壤污染状况详查，深入推进“清废”行动，解决固废、危废问题584个，妥善处理了高义钢铁违法倾倒钢铁废渣危及环境安全问题。全面推进国土绿化，实施南山百里绿廊和盐湖十里北坡绿化工程，完成造林17.8万亩。

这一年，我们倾力保障和改善民生，大力发展社会民生事业，人民群众的获得感幸福感安全感有效提升。民生投入力度持续加大。财政资金用于民生领域支出达280.1亿元，占到一般公共预算支出的82.9%。脱贫攻坚取得决定性进展。平陆、万荣、垣曲进入摘帽退出程序，完成181个贫困村退出，7.7万人脱贫，贫困发生率降至0.55%；新建的36个易地扶贫搬迁集中安置点全部竣工，搬迁1.7万人，“十三五”易地搬迁任务全面完成，有效解决2.6万贫困人口饮水安全问题，易地扶贫搬迁和农村饮水安全两个省级现场会先后在我市召开。社会保障不断加强。城乡低保标准每人每月提高45元，比省定任务翻一番；农村低保平均标准为4067元/年，高于全省标准667元；城乡居民基本养老保险基础养老金增长28.8%。新建农村老年日间照料中心219个，累计1314个。全市开通跨省异地就医直接结算医疗机构33家。教育事业扎实推进。新（改、扩）建22所公办幼儿园，认定155所普惠性民办幼儿园。高中教育质量稳步提升，高考应届生一本二本达线人数分别增长12.4%和8.9%。职业教育培养技能型人才3万余人。体育工作成绩斐然，省运会上取得30年来最好成绩。就业工作卓有成效。转移农村劳动力8.6万人，城镇新增就业6.2万人，城镇登记失业率控制在2.31%。民生实事顺利推进。省、市确定的11件民生实事全面达到预期目标，人民群众得到切实的便利和实惠。社会治安和安全生产形势稳定。扫黑除恶专项斗争战果丰硕，全年共打击涉黑涉恶犯罪团伙100个、村霸26个、恶霸10个；各类刑事案件、治安案件同比下降17.6%、20.4%。全年安全生产事故起数和死亡人数均实现大幅下降，社会保持和谐稳定。

这一年，我们坚持依法行政，深化“放管服效”改革，政府自身建设不断加强。党风廉政建设进一步强化。认真落实全面从严治党主体责任，深入推进政府系统党风廉政建设和反腐败工作，努力营造良好的政治生态。依法行政深入推进。严格重大行政决策合法性审查，积极配合推进人大立法工作，政府法律顾问实现市县乡三级全覆盖。主动接受人大、政协监督，全年办理市人大代表建议318件、政协委员提案274件，办复率和满意率均达100%。“放管服效”改革持续深化。市本级在承接省政府取消、清理规范的106项行政职权事项基础上，自主取消下放62项行政职权，并向开发区下放242项行政审批事项。市县两级政务服务中心全面建成并规范化运行，初步实现了“四办”目标，前置审批事项由226项削减为63项，进一步激发了市场活力，全市新登记市场主体5.7万户。“12345”政务热线实现24小时服务，受理热线电话32611个，转办事项办结率99.7%、群众满意率99.99%。同时，国防建设、军民融合持续加强，省级双拥模范城创建顺利通过。统计、人防、档案、地方志、民族宗教、防灾减灾等工作不断加强，妇女、儿童、老龄、残疾人等各项社会事业也取得新成绩。

各位代表，过去一年，我们认真分析、准确把握国际国内形势，以开放的思维谋求发展，以创新的理念推动转型，以务实的举措改善民生，成绩来之不易。这些成果，靠的是习近平新时代中国特色社会主义思想的指引，靠的是党中央、国务院和省委、省政府的科学决策部署，靠的是市委统揽全局、坚强领导，靠的是市人大、市政协的有效监督和鼎力支持，靠的是全市广大干部群众团结拼搏、共同奋斗。在此，我代表市人民政府，向全市人民，向各民主党派、工商联和无党派人士，向各位人大代表、政协委员，向驻运部队、武警官兵和公安干警，向所有关心支持运城改革发展的各界朋友，表示崇高的敬意和衷心的感谢！

一年来，我们在实践中深化了对做好新形势下政府工作的规律性认识：必须坚持党的集中统一领导，提高政治站位，坚定不移推动中央、省市各项决策部署落地见效；必须坚持转型主线和高质量发展要求，持续不断优化经济结构，坚定不移走无煤少煤市转型发展新路子；必须坚持深化改革开放，着力破解发展瓶颈，坚定不移打造对外开放新高地；必须坚持环保倒逼，强化绿色发展理念，坚决打好污染防治攻坚战，坚定不移推进生态文明建设；必须坚持以人民为中心，切实保障和改善民生，坚定不移把增进民生福祉作为改革发展的出发点和落脚点，不断增强人民群众的获得感幸福感安全感。

同时，我们也清醒地看到，全市经济社会发展中仍面临着突出矛盾和问题：一是高质量转型发展任务艰巨。三次产业结构不优，实体经济实力不强，产业结构偏重，发展方式粗放，大多处于产业链中低端，长期积累的结构性、体制性、素质性矛盾还未得到根本解决。二是环保压力较大。污染防治形势严峻，一些地方和部门还不能正确处理环境保护与经济发展的关系，抓环保力度存在层层递减的现象，特别是我市纳入汾渭平原大气污染防治重点区域后，工作要求更高、挑战更大。三是风险防控难度加大。预防和应对经济社会等各种风险意识还不强、能力还不足，金融担保、政府债务、信访维稳、安全生产等领域风险点较多，对我们工作提出了新的挑战。四是项目建设支撑能力不足。全市上下谋项目、抓项目的氛围还不够浓厚，力度还不够大，开发区引领作用还不够突出，大项目、好项目还不多，转型发展缺乏强有力的项目支撑。五是营商环境还需下大气力优化。个别干部作风不实，抓落实力度还不够，少数部门和单位依然存在中梗阻现象，不作为、慢作为问题还未得到彻底解决，行政效能有待进一步提高。六是创新人才短缺。适应高质量转型发展、市场竞争、改革开放等内在要求的高端人才、专业化人才还很匮乏，人才引进渠道还需拓宽，人才创业环境还需优化，人才激励机制还需持续完善。对此，我们将坚持问题导向、目标导向，采取更加有力举措，努力加以解决。

二、2019年工作安排

2019年是中华人民共和国成立70周年，是全面建成小康社会的关键之年，是我市在“两转”基础上拓展新局面的攻坚之年。今年政府工作的总体要求是：以习近平新时代中国特色社会主义思想为指导，全面贯彻党的十九大和十九届二中、三中全会精神，统筹推进“五位一体”总体布局，协调推进“四个全面”战略布局，深入贯彻习近平总书记视察山西重要讲话精神，坚持党的领导，坚持稳中求进工作总基调，坚持新发展理念，扎实开展“改革创新、奋发有为”大讨论；围绕省委“示范区”“排头兵”“新高地”三大目标，以“改革抢先机，发展站前列，各项工作创一流”为总要求，以“走进新时代，建设大运城”为总抓手，落实“一区五带”发展布局，扭住“转型综改、改革开放、实体经济、项目建设、生态环保、民生改善”六个重点，继续打好“三大攻坚战”，进一步稳就业、稳金融、稳外贸、稳外资、稳投资、稳预期，巩固和拓展转型发展、高质量发展的好势头，为全面建成小康社会收官打下决定性基础，以优异成绩迎接中华人民共和国成立70周年。

主要预期指标是：全市地区生产总值增长6.6%左右，固定资产投资增长6.5%，社会消费品零售总额增长7.5%，一般公共预算收入增长10%，城乡居民人均可支配收入分别增长6.5%和7%以上，居民消费价格涨幅控制在3%左右，城镇新增就业5万人，城镇登记失业率控制在4.2%以内。约束性指标不折不扣完成省定任务。

实现上述目标，今年要重点抓好以下工作：

（一）坚持稳中求进总基调，确保“六稳”基础上的新跃升

按照党中央坚持稳中求进工作总基调和做好“六稳”工作的战略部署，贯彻落实“巩固、增强、提升、畅通”八字方针，着力在“稳”字上下功夫，在“进”字上求突破；保持战略定力，坚持转型发展主线和高质量发展要求，强化创新驱动发展战略，在走好运城发展三条新路子上实现新突破；增强危机意识，积极应对经济形势稳中有变、变中有忧的新态势，在复杂的形势变化中抢先机、赢主动、保发展。

加强“一区五带”规划引领。全面落实“一区五带”发展布局，牢固树立全市发展“一盘棋”思想，明确县域产业定位和主攻方向，优化资源配置，加强区域协调，突出特色化、差异化发展，推动市域经济板块特色更加鲜明，产业聚集度显著提高，主体功能区作用充分发挥，引领全市经济持续健康发展。

加强经济运行调度和服务。密切关注宏观经济主要指标和先行指标变化情况，加强对重点行业、重点企业、重点产品的运行监测，强化要素协调，及时解决经济运行中存在的困难和问题，全面落实承诺制、末位解剖制和月调度机制，压实责任，提升效能，确保如期完成经济社会发展既定目标。

加强项目谋划和招商引资。强化重点项目深度谋划，依托当地资源禀赋优势，制作区域经济、行业领域的项目图谱，引领项目建设。强化招商引资，进一步优化开发区招商引资体制，搭平台、建机制、抓队伍，明确招商重点与路径，全面提升招商引资成效。

加强重点项目建设。围绕全省深化转型项目建设年活动，深入推进“3515重大工程项目”“龙腾虎跃千亿工程”“城建111工程”等重点项目建设，推动总投资4013.6亿元的725个重大转型项目按期开工建设、投产达效。抓好大运汽车纯电动轻型客车及汽车产业园、垣曲抽水蓄能电站、芮城光伏领跑二期、山西建龙140万吨冷轧卷板、阳煤丰喜100万吨甲醇和40万吨乙醇、中材集团运城超低能耗建筑产业园，以及市会展中心和农产品仓储物流园等重大转型项目，不断培育新的经济增长点；抓好运城国际机场扩建、动车存车场扩建、蒙华铁路运城段、临猗黄河大桥及引线工程、运三高速三门峡公铁黄河大桥等重大基础设施项目，积极做好运三客运专线、阳运高速公路和韩城－河津－侯马城际铁路前期工作，夯实发展基础；抓好解放路和圣惠路提升改造、中银大道、城市水系、市民服务中心、文化艺术中心、池神庙文化广场等市政项目，提升城市品位和形象；抓好中恒运城文化教育产业园、盐湖康养度假区、夏县嫘祖文化产业园颐养园、中小学幼儿园等社会民生项目，不断满足人民群众美好生活需要。我们就是要严格落实“四个一”项目推进机制，加强项目调度分析，加快项目落地见效，全面推动重大项目建设实现新突破！

（二）坚持创新驱动路径，构建先进制造业为先导的现代工业体系

深化供给侧结构性改革。压减淘汰落后产能，大力推进焦化、钢铁等传统产业工艺技术改造、生产线优化升级、环保特别排放和超低排放改造，在绿色发展上全面跃升新台阶。大力推进产业链延伸和产品提质，通过技改专项行动和新产品研发专项行动，促进原材料产业向中下游深加工延伸、整机产品向中上游延伸，实现市域范围内的关联产业融合配套发展。支持生产型企业向创新型企业发展，不断增强企业自主设计、自主研发能力，遴选并推进100个重点技改项目跃升新水平，着力培育100个重点新产品开拓新局面。

加快“三大基地”建设。中西部精品钢基地建设上，围绕钢铁产业改造升级和产业链延伸，着力打造以山西建龙为核心的闻喜精品钢产业园和新绛高义、河津宏达两个精品钢园区，加快关联产业入园，形成集聚效应，力争形成500亿元规模的精品钢产业板块。国家级铝镁合金产业基地建设上，针对中高端铝镁合金产品市场需求，以深化电力改革、铝电协同优化为突破，加快煤电铝材一体化步伐，推动“一中心两试点五园区”建设，延伸铝镁合金深加工产业链条。抓好复晟铝业120万吨氧化铝、云海铝业中间合金等10个重大项目，力争形成300亿元规模的铝镁合金产业板块。汽车和新能源汽车基地建设上，以大运汽车为核心，建设园中园，推进以企招商，力争吸引一批关联企业入驻。重点抓好大运汽车10万辆电动SUV、山西建龙汽车用钢、亚新科发动机零部件智能化加工、阳煤千军发动机缸盖生产线扩建等项目，形成整车生产、零部件配套、检验检测等较为完整的产业体系，力争形成300亿元规模的汽车与新能源汽车产业板块。

打造“四大产业链条”。汽车产业链条上，以大运汽车为核心，发展汽车轮毂、轮胎、面板、控制平台等配件产业；以诺维兰为主体，发展汽车生产性服务业，建设汽车服务综合体、汽车文化广场和营销服务网络，打造汽车工业生态链、产业圈，实现链条化、集群化发展。铝镁合金产业链条上，加强科技研发，突破技术瓶颈，强化氧化铝就地转化，加强电解铝深加工，推广轻量化应用，加速向工业中间品、3C产品、航空航天产品、高端制造、智能家居产品等领域延伸，构建产业联盟，增强产业协同，不断提高铝镁合金产品附加值和整体竞争力。医药康养产业链条上，打好特色医疗牌，发挥好83万亩中药材种植基地、19家规上医药企业及440家各类医疗机构的资源优势，强化上下游合作，研制新产品，促进中医中药、中成药、仿制药品产业化，建设特色医疗城，打造中医药强市；充分挖掘盐湖黑泥、温泉等康养资源，建设一批康养小镇、康养度假村，带动康养产业发展。农产品深加工产业链条上，紧盯国际国内功能农业发展趋势，充分利用好我市丰富的农业资源优势，延长产业链、提升价值链、优化供应链、构建利益链。加大苹果、山楂、樱桃、葡萄、芦笋、红枣、核桃等特色农产品深加工精准招商和资源整合，进一步丰富品种、提升质量、创建品牌，形成种类多元、特色鲜明、品牌响亮的农产品深加工产业体系。全市主要农产品加工转化率达到60％以上，年加工销售收入达到320亿元。

培育壮大新兴产业集群。装备制造产业，重点发展汽车、机电、化工、军工等领域装备产业，着力推动大运汽车、永济电机、运城制版、阳煤丰喜、国澳崇基、中信机电等一批重点企业装备产业园区建设，促进产业高端化、规模化发展。新材料产业，发挥我市在铝镁合金、精品铜、稀土永磁、纳米材料、净水材料、电子信息、工程塑料等材料领域基础优势，加强与国内外知名科研机构合作，不断优化提升技术水平，拓展应用领域，打造重点领域国家级创新与产业化基地。新能源产业，推进能源体制改革，着力推动光伏、风电、抽水蓄能、生物质发电等重点项目建设，深化铝镁、钢铁等重点领域节能改造，争做能源革命排头兵。电子信息产业，坚持应用牵引，大力发展大数据、云计算、移动互联、物联网、人工智能以及电子元器件、电子产品组装、整机制造及应用等新一代信息技术产业；大力拓展4G网络全覆盖和5G网络试点，依托大数据中心，深化与互联网新经济战略合作，支持电信运营商、寰烁科技、国强高科、运城中兴等重点企业本地化发展，培育壮大信息产业新主体。通用航空产业，抢抓国家低空开放重大政策机遇，加快推进芮城、万荣、绛县等通用机场建设，发展通用航空装备制造，拓展环境监查、空中巡查、应急救援、短途运输等应用服务，培植新的经济增长点。

深化创新驱动战略。大力推进高新技术企业、民营科技企业和科技型中小企业发展。支持企业技术中心等创新平台建设，力争年内新增2－3家院士工作站和2－3家博士后科研工作站。依托龙头企业，重点攻关铝镁合金、精品钢、轨道交通、机电、新能源汽车、生物医药、F型杂交小麦等技术瓶颈，掌握一批核心关键技术，促进产业提档升级。加强与高校、科研院所及创新型企业合作，积极开展科技成果入企活动，打通转化“最后一公里”。落实“三晋英才”支持计划，加大科技领军人才培养和引进力度，落实好人才激励政策，努力打造一支本土化创新人才队伍。坚持市场导向，引导在运高校专业调整设置，培养转型发展适用人才。继续实施“两化”深度融合和智能制造三年专项行动，遴选50户重点企业建立健全“两化”融合体系，达到贯标标准，20户重点企业实施智能制造提升，实现生产设备智能化、生产过程自动化、企业管理信息化和供应链条网络化。加速“互联网+工业”体系建设，抓好“企业上云”，促进先进制造套件服务和在线信息服务新模式创新，有效提升企业数字化生产、柔性生产和弹性制造能力。

推进先进制造业与现代服务业深度融合。加快服务业集聚区建设，培育一批省级示范园区。开展服务型制造示范，促进生产型制造向服务型制造转变。大力发展生产性服务业，以山西建龙为试点，创新金融服务，发展废钢加工配送。加快现代物流服务业，重点推进农产品交易中心、黄河金三角国际果蔬物流港等重大项目，积极发展供应链物流、智慧物流、冷链物流。

各位代表，构建现代工业体系是事关我市高质量转型发展的长远大计。我们必须横下决心，举全市之力推动产业技术变革和结构优化升级，奋力走出运城工业经济高质量发展新路子！

（三）坚持乡村振兴战略统领，加速农业农村现代化步伐

贯彻落实中央一号文件精神，围绕“大力发展现代农业、全面深化农村改革、建设美丽乡村、脱贫攻坚”乡村振兴四大任务，突出抓好“农民增收、农业基础设施建设、农业优质品牌打造、大规模植树造林、农村改革、农村人居环境整治”六个重点，巩固持续向好形势，推进农业农村全面振兴。

大力推进农业产业体系特色化、融合化发展。依托黄河经济带、峨嵋岭经济带、中条山经济带的区位、资源和生态优势，引进大型深加工企业，着力打造运城优质面粉、优质水果、优质蔬菜、优质功能食品等特色产业。成立市级农产品深加工产业集团，设立产品培育基金，扶持晋龙、天之润、格瑞特、运康锅巴等企业做大做强，形成一批在全国有影响力和市场占有率的知名产品。以“一区三园”创建为抓手，再规划打造5个现代农业产业园。发展以农业资源为牵引的一二三产融合发展新业态，着力建设特色优势产业发展区，打造6个农旅康养示范区、6个功能农业示范区、5个商贸物流示范区、3个城郊农业示范区、2个农产品加工示范区，培育产业兴旺新主体。

大力推进农业生产体系标准化、基地化发展。大力实施农业标准化战略，加大“三品一标”认证力度，发挥省级优质农产品出口平台的导向作用，坚持标准倒逼，提升现代农业标准化生产水平，新建20个标准化果园、20个标准化蔬菜园、20个标准化干果园，新增标准化生产面积60万亩，积极培育区域“名优特”果品生产基地。发展智能农业、循环农业、设施农业，强化现代农业生产要素优化配置，打造50个高标准农业科技创新示范基地，形成示范带动效应。加快农机装备升级，积极推进农田“宜机化”改造，着力提升丘陵山区农业机械化水平和果业、蔬菜、药材的机械化水平。大力推进省级农机化综合示范市试点创建。大力支持农业科技创新和科研成果推广应用，推广一批集成化集约化新技术，推动农业全产业链改造升级。

大力推进农业经营体系组织化、市场化发展。培育壮大农民专业合作社等新型经营主体，大力发展“基地+企业+合作社+农户”的生产组织与经营模式，提升农业生产效益。发展壮大新型农村集体经济，逐步消除集体收入5万元以下的薄弱村。大力发展“互联网+农业”，推进智慧农业云平台建设，发展农业电商，提升互联网精准营销水平，拓宽农产品销售渠道。狠抓农产品质量安全监管，加强农产品检验检测，倒逼农产品质量提档升级。强化“运城苹果网”建设，深化苹果价格指数应用，扩大“运城苹果”品牌影响力，提升运城果业竞争力。

大力推进现代农业有机化、节约化发展。深入实施化肥零增长行动，推进农药减量行动，加强高效植保机械和新农药新技术推广应用，加速有机肥替代。推广耕地土壤改良，推进有机旱作节水农业，规划创建一批绿色、高质、高效旱作节水农业示范片区，再建高标准农田50万亩。强化农田水利建设，加速小浪底引黄、部官引黄等重大水利工程建设，完善末级渠系配套，进一步扩大引黄灌溉覆盖面。

大力推进现代农业循环化、无害化发展。着力发展规模养殖，做好牧原、温氏、大象、长荣等项目拓展，发展晋南大黄牛等优质品种和特色水产养殖，提升畜牧业供给能力和经济贡献率。积极推广秸秆直接还田、燃料化、饲料化等循环化利用，强化重大动物疫病防控体系建设，抓好畜禽废弃物资源化和无害化处理。

大力推进现代农民专业化、职业化发展。着眼谁来种地和现代农业发展，培养一批懂农业、爱农村、爱农民的干部队伍和新型农民。大力推广“中农乐模式”，推进与国内知名农业科研院所合作，开展现代农业科技知识普及，年内再完成10万农民技能提升，培养专业化职业农民1万人。

大力推进农村人居环境改善。落实农村人居环境整治三年行动实施方案，扎实开展拆违治乱、生活垃圾治理、生活污水治理、厕所革命、卫生乡村建设五大专项行动，深入开展“五星级文明户”和“四好卫生家庭”创建活动，年底前各县（市、区）全部建立农村生活垃圾收运处理体系。抓好农村绿化美化净化硬化工作，启动建设200个市级以上城郊融合、文化旅游、休闲养生、农耕体验、绿色生态等不同类型的美丽乡村。加大传统村落保护力度。抓好“大棚房”问题专项清理整治，坚决防止“农地非农化”倾向。

大力推进农村改革。进一步巩固和完善农村基本经营制度，推进农业生产托管服务国家级试点工作，稳妥推进土地规模流转，全年新发展农民合作社400个、培育省级示范社35个；深化农村土地制度改革，做好土地确权信息管理及成果转化应用；深化农村集体产权制度改革，积极探索集体资产股权质押贷款办法，推动农村各类产权流转交易公开规范运行；完善农业支持保护制度，健全农业保险保障体系，设立农业发展基金，引导社会资本进入涉农产业、企业和经营主体；加快农业科技体制改革，鼓励科技人员下乡，支持科技人员与农民合作推进技术革新，并参与科技成果转化增量收益分配改革；强化党建引领，加快构建自治、法治、德治“三治融合”的乡村治理体系；完善脱贫攻坚与乡村振兴有机衔接的政策体系，筑牢返贫防线，提高脱贫质量；健全支持有机旱作农业和城郊农业发展的有关政策，打造示范片区，促进农业生产能力提档升级。

各位代表，做好“三农”工作是全面建成小康社会的“压舱石”。我们将始终坚持农业农村优先发展，深入实施乡村振兴战略，全力推动“八大提档升级行动”，让农业农村现代化成为运城最大的亮点！

（四）坚持以融合发展为纽带，打造全域旅游目的地

构建全域旅游发展新格局。挖掘“六大文化”资源和丰富的自然资源，紧扣“古中国”“关公”两大知名品牌，聘请国际化专业团队，统筹谋划“四大旅游板块”，打造20个品牌景点，推出6条精品线路，在黄河、太行两大主题上实现新发展。加强线上线下旅游目的地宣传推介活动，增强与重点区域的对接合作，不断提升运城全域旅游的吸引力。

推动景区提档升级。加快关帝庙、李家大院、鹳雀楼等创建国家5A级景区，力争两年内实现零的突破；积极推进关圣文化建筑群申遗工作，打造解州历史文化名镇。全力推动671公里沿黄旅游公路建设，抓好薛仁贵故里、普救寺婚礼殿堂、圣天湖康养度假区、周仓文化园、祁家河风景区和曙猿湾房车宿营地等重点项目建设，打造黄河全景慢游走廊和自驾游目的地。

完善旅游配套服务。围绕“吃、住、行、游、购、娱”六要素，强化旅游业软硬件配套服务。加快市、县两级旅游集散中心和旅游咨询服务中心建设，开通市域重点景区免费巴士，开发特色文旅产品，培养高水平导游队伍。加快发展智慧旅游，构建旅游大数据支撑平台、智慧旅游综合服务平台，为游客提供一站式套餐服务，全面提升旅游服务质量。

提升公共文化服务水平。培育和践行社会主义核心价值观，深入实施公民道德建设工程。推动基本公共服务标准化均等化，加强县级文化馆、图书馆总分馆制建设和基层综合性文化服务中心建设。深入推进文明守望工程、乡村文化记忆工程和文化惠民工程，创新文物保护利用机制，加大文物保护力度，推进文物密集区体制改革试点工作，积极申报第八批国保单位。大力支持文艺精品创作，积极开展对外文化交流，为人民群众提供更多更好的精神食粮。

各位代表，文化旅游业是我市开放发展的新载体、转型发展的突破口、高质量发展的新引擎。我们将继续办好“三节三会”，叫响运城旅游品牌，扩大“古中国·大运城”的影响力，真正把文化旅游业打造成我市的战略性支柱产业！

（五）坚持振兴实体经济不动摇，不断增强市域经济竞争力

深入推进“三个发展计划”。“龙腾虎跃”计划方面，“龙榜”企业要在产值规模、经济效益、市场占有率上进一步巩固，在技术创新能力、管理能力、产业链协同能力和市场应变能力上进一步增强，在园区化、集群化、集聚化上进一步拓展，力争年内“龙榜”企业达到5家。“虎榜”企业要在发展基础、专业化能力上巩固，在项目建设、新产品培育上增强，在市场空间和资源合作上拓展，在运行机制和发展模式上创新，加快成长步伐，年内“虎榜”企业力争达到40家。“群星灿烂”计划方面，实施“小升规”专项行动，制定优惠政策，设立“新秀奖”，优先考虑基金和项目支持，年内新增80家“小升规”企业，完善提升10家中小微企业公共服务平台；大力支持“双创”基地创建，年内新建10－15个“双创”基地，对入驻企业给予奖励；以“四新”为主的重点项目力争完成投资100亿；举办第二届工博会，设立产融交易、品牌推介、人才招聘等专场活动，为企业发展搭建平台、创造商机。“凤还巢”计划方面，健全主要城市活动阵地，搭建合作交流平台，促进产业拓展；开辟“凤还巢”集聚区和创业园，设立“凤还巢”专项基金，支持创新创业。

深化民营经济改革“五项工程”。民营企业改制工程上，大力度推动民营企业建立现代企业制度，年内推进50家民营企业改制示范。民营经济管理提升工程上，以“龙虎榜”培育企业为重点，优选100家民营企业引领带动，提升企业现代化管理水平。民营企业资产证券化工程上，加快大运汽车、阳光焦化、寰烁科技、豪钢锻造等企业上市步伐，力争1家企业向证监会申报IPO，5家企业在“晋兴板”挂牌，50家企业纳入上市挂牌后备企业资源库。民营企业信用等级评定认证工程上，加快建立和完善企业信用体系建设保障机制、监管机制，培育和规范企业信用市场，强化企业信用自律管理，力争年内完成100家民营企业信用评定。民营经济品牌战略工程上，开展工业企业品牌培育试点，打造一批区域经济带动性强、市场拓展空间大、质量诚信度高的示范企业；举办“运城市品牌发展水平（成果）评价活动”，对优质品牌进行表彰、奖励、扶持。

落实支持实体经济发展壮大的政策举措。落实中央、省支持民营企业发展壮大各项政策举措，制定出台我市支持政策。“一企一策”帮助企业解决生产经营难题。特别是要多措并举，畅通中小企业融资渠道，有效解决企业融资难融资贵问题。搭建民营企业与金融机构对接机制，切实解决信息不对称问题，夯实融资基础；健全地方融资担保机制，为企业融资提供支撑；加大基金运作力度，整合社会资本，支持重点企业、重点项目建设；加快企业信用体系建设，为企业融资提供信用保证。

各位代表，实体经济是运城振兴的基础，民营企业是运城崛起的依托。我们要不折不扣落实好国家、省各项政策措施，下大力气破除制约发展的各种障碍，解除企业家的后顾之忧，把牢运城经济高质量转型发展的“定盘星”！

（六）坚持深化改革扩大开放，充分释放发展活力

准确把握市场化改革要求，创新体制机制，推动关键领域改革走深走实。深化医药卫生体制改革。推动国家中医药综合改革试验区申报工作。学习借鉴先进地区先进做法，巩固提升县域综合医改成果，深化盐湖、万荣、河津示范区打造，在一体化、集团化、信息化、均等化、便民化上下功夫，切实解决群众看病难、看病贵问题，确保全市域医改工作继续走在全省、全国前列。深化开发区改革。全面推进“三化三制”改革，达到全省领先水平。全力推进管运分离，建设园中园，实现产业聚集。加快推进运城经济技术开发区、盐湖高新技术产业开发区申报国家级经济技术开发区和国家级高新区工作，力争年内获批。突出重大项目牵引，加强投资强度、产出强度、税收强度考核，开展开发区土地利用节约集约评价，推动开发区提质升级。深化国资国企改革。积极推进处僵治困，实施僵尸企业3年出清计划，完成黄金工业公司、化学工业公司等6家企业改制出清工作，完成国有企业“三供一业”维修改造工作。

深化教育体制改革。抓紧做好高考制度改革各项准备工作，确保高考质量稳步提高。抓好中小学优质教育资源均等化改革，推动山区和力量薄弱地区教学质量有效提升。同时，抓好政府机构、财税体制、金融体制等改革，确保严格按照时间要求、任务要求改革到位。

牢固树立开放发展理念，持续打好对外开放“七张牌”，推动形成东融西进南联北拓的开放新格局，构建内陆地区对外开放新高地。加强开放平台建设。瞄准区域性中心国际机场、区域性航空货运枢纽发展目标，同步推进自贸区建设和航空口岸正式开放，实现贸易经济和临空经济深度融合。培育壮大外贸主体。实施对外贸易主体培育三年行动计划，建立市级外贸企业孵化中心，扶持外贸主体“小升规”，支持玻璃器皿、铝镁合金、纺织面料等重点外贸企业对外发展。深化农产品出口“三个100”，扩大特色优质农产品出口。深化区域合作。用好长三角考察学习成果，加强同京津冀、环渤海、长江经济带的交流合作，鼓励各县（市、区）、开发区与长三角、珠三角等发达地区的县（市）和开发区开展战略合作，积极承接产业转移，加快产业聚集，实现弯道、换道超车。深化晋陕豫黄河金三角区域合作，加快推进一批省际交通基础设施、生态治理试点等跨区域重大项目。扩大国际合作。积极对接参与“一带一路”建设，扩大与沿线国家的贸易往来，引导和支持运城制版等更多企业参与国际竞争。积极参加国际性博览会和中国进口博览会，进一步拓展建立2—3个国际友好城市，提升国际化水平。

（七）坚持大运城规划引领，加快新型城镇化进程

坚持规划引领。高标准完成2035版城市总体规划、北部新城片区规划、南部盐湖生态文化区规划、官道河生态水系规划编制及实施工作，进一步拓展城市发展空间。

建设高品质城市。统筹推进中心城区及各县城基础设施、产业布局和公共服务提升，统筹地上与地下、新区与老城区建设，集中实施一批重大项目，推动城市建设实现新突破。加快中心城区东部片区和姚孟片区开发，加大“城建111工程”建设力度，完善城市路网，加快机场到高铁站道路建设，推进解放路和圣惠路提升改造，打通河东西街延长线，缓解交通压力。规划打造休闲、餐饮、商贸等功能街区，加强商业综合体建设，满足不同层次的消费需求。加快推进城市公共停车场、垃圾中转站、雨水泵站等市政公用设施建设。加强城市绿化美化亮化，建设植物园，完善提升小游园。加大力度推进棚户区、城中村改造，推进保障性住房建设，促进农村人口向城市转移。

提升城市管理水平。以创建全国文明城市为牵引，统筹推进“五城同创”。深化智慧城市“一平台十应用”，持续建设宽带运城、数字运城、智慧运城。推动居住证制度全覆盖，提高户籍人口城镇化率。强化城管文明执法，改善城市环境卫生，增强市民意识，规范交通秩序，提高城市网格化、精细化管理水平，打造生态宜居、整洁便民的城市环境，推动城市管理实现根本性改变。

加快城乡一体化步伐。积极推进撤县设区，全面做好夏县撤县设区获批后各项工作，稳妥做好临猗撤县设区以及闻喜撤县设市相关工作。稳步推进撤乡设镇，做好河津市柴家乡撤乡设镇相关工作，促进城乡一体融合发展。加快推进特色小镇建设，合理安排产业集聚、村落分布等空间布局，推动基础设施城乡联网、共建共享，着力培育一批经济繁荣、环境优美、辐射力强的特色小镇，将“一主三副七个大县城71个建制镇”城镇化工作引向深入。

（八）坚持生态文明思想，坚决打赢打好“三大保卫战”

坚决打赢蓝天保卫战。严格落实运城市打赢蓝天保卫战三年行动计划和2018—2019年秋冬季污染防治攻坚战各项任务。抓好关铝热电供热机组技术改造，持续推进中心城区和平原农村地区冬季清洁取暖工作，年内完成清洁取暖20万户。全面开展“散乱污”企业整治，深入排查挥发性有机物排放，加强柴油车管控，加强扬尘综合治理。科学制定差别化停产限产措施，优化企业环境治理方案，避免“一刀切”。

坚决打好碧水保卫战。紧盯国考断面水质达标，抓好城镇污水处理厂保温提效、配套管网工程建设。加大“三河三湖”生态修复治理力度，加快新绛、稷山、河津城区及汾河入黄口生态湿地建设，推进汾河沿线生态修复和农村污水处理厂等重点工程，加强水源地保护，加快涑水河入黄段水生态治理。深入推进城市黑臭水体治理，实施雨污分流改造，中心城区和各县城建成区黑臭水体消除比例要达到90%以上。

坚决打好净土保卫战。强化农用地土壤和建设用地环境管理，严格管控土壤环境风险，推进生活垃圾减量化、资源化和无害化处理。持续开展“清废”行动，加强危废规范化管理，提升危废处置能力，破解工业固废历史堆存难题，从源头上推进固体废弃物减量化。

抓好植树造林和绿化工作。启动国家森林城市创建工作，全民发动，加大投入，集中开展植树造林，完成20万亩造林任务。每个县（市、区）高标准完成省道、市道和县道主要道路通道绿化提档升级。抓好荒山荒坡和乡村庭院绿化，继续实施南山百里绿廊和盐湖十里北坡绿化工程，让绿色成为运城发展底色。

（九）坚持以人民为中心发展思想，着力改善民生福祉

坚决打好精准脱贫攻坚战。筑牢返贫防线，着力巩固提升脱贫攻坚成果，做到脱贫不脱政策、脱贫不脱责任。做好易地搬迁后续工作，提高入住率和拆除复垦率。加强干部驻村帮扶和产业支撑，扶志扶智相结合，激发贫困群众内生动力，确保剩余2.3万贫困人口全部脱贫。探索建立返贫预警机制，实施动态管理常态化，做到应纳尽纳、返贫即入、应扶尽扶、脱贫即出。

促进就业创业。发挥创业带动就业的倍增效应，实施“双创”引领，促进就业，吸引创业。选树运城特色劳务品牌，推进劳务输出品牌化发展。加强就业形势监测，做好高校毕业生、退役军人、转岗安置职工、农民工、就业困难人员等群体就业工作，强化就业服务，确保零就业家庭动态清零。

完善社会保障。积极推进全民参保，促进应参尽参、应保尽保。做好机关事业养老保险与企业职工养老保险并轨运行，构建覆盖城乡的社会保障体系。

提高教育质量。实施学前教育普惠行动，深入推进城乡义务教育一体化发展，深化城镇教师支援农村教育工作，推动基础教育优质均衡发展。加快职业教育提质增效，支持和规范民办教育，巩固校外培训机构整顿成果，推进智慧教育二期工程，全面提升教育综合实力。大力推进素质教育，促进学生全面发展、健康成长。

大力发展医疗卫生事业。着力培育具有示范带动作用的医疗专科和学科带头人，构建10个市级中医重点专科。做实做细家庭医生签约服务，确保签约率稳步提升。启动市中心医院二期工程，加快特色医疗城项目建设。加强预防保健和妇幼保健工作，提高公共卫生服务水平。加快发展大健康产业，打造“医、养、健、管、游、食”大健康全产业链，优先使用基本药物，降低群众用药负担，方便群众就医，为群众提供全方位全周期健康服务。

完善住房保障体系。坚持“房住不炒”定位，大力发展住房租赁市场，落实住房租赁税收优惠和金融支持政策。加快推进农村危房改造。大力发展装配式建筑，提高全装修住宅覆盖率。建立健全房地产市场调控长效机制，保持房地产市场稳定，让住房保障政策惠及更多城镇中等及以下收入家庭。

各位代表，今年我省将举办第二届全国青年运动会，我市承担着圣火采集、火炬传递和部分赛事承办的重要职责。我们要举全市之力，精心组织，全力做好各项筹备工作，圆满完成省委、省政府交给我们的重大任务。同时，借此机会，大力宣传运城发展成就和对外开放形象，不断提升运城美誉度和知名度！

各位代表，我们要持续办好为民实事。今年我们将全面承接、认真办好省政府确定的八件民生实事：一是继续实施全民技能提升工程，再培训10万人；二是将残疾预防重点干预和残疾儿童抢救性康复项目帮助对象由6025名增加到7285名；三是继续为全部贫困县妇女实施免费“两癌”检查服务；四是继续为怀孕妇女提供免费产前筛查与诊断服务；五是再新建60个日间照料中心；六是继续免费送戏下乡2000场；七是实施经济困难的高龄和失能老年人关爱行动工程；八是实施免费法律咨询便民工程。在此基础上，全市还将办好两件民生实事：一是实施中心城区和各县城小街小巷改造提升工程，年内中心城区完成160条。二是实施中心城区和各县城公厕建设工程，年内中心城区新建成20个。民生实事连着千家万户，我们就是要坚持人民立场，用非常之力，下恒久之功，把实事办实、好事办好，不断增强人民群众的获得感、幸福感！

（十）坚持底线思维，防范化解重大风险

加强应急管理和安全生产。全面深化应急体制改革，开展安全生产大排查大整治，深化重点行业、重点领域专项整治，确保安全生产形势持续稳定好转。健全完善应急管理体系和应急预案体系，强化应急救援队伍建设，加强实战化应急演练，做好各项应急准备工作。加强气象、地震、人防等工作，强化灾害风险防范措施，全面提高综合防灾减灾救灾能力。严防突发环境事件、重大食品药品安全事故、重大交通事故等公共安全事件，打击非法超限超载运输行为，坚决杜绝重特大事故。

优化金融生态环境。加强金融机构监管，支持金融创新，进一步加大金融服务实体经济力度。落实属地责任，“一企一策”化解担保链风险。提高风险监测和预警能力，严厉打击恶意逃废金融债务行为，打击非法融资活动，牢牢守住不发生系统性金融风险和经济风险的底线。

强化信访维稳。全面落实总体国家安全观，提高风险预知预警预判能力，紧盯重大敏感节点，做到“零懈怠”“零疏漏”“零失误”。完善社会矛盾纠纷多元化解机制，创新信访工作方式，做实做细做好各类利益诉求群体的信访维稳工作。

深化社会治理。持续推进扫黑除恶专项斗争，着力在深入打击农村黑恶犯罪、巩固基层政权、深挖“保护伞”、摧毁黑恶经济基础、铲除黑恶势力土壤上出硬招、见实效。做好民族宗教工作，支持国防建设，加强“双拥”工作。更好发挥工会、共青团、妇联等群团组织作用，加强城乡社区治理，发挥社会组织作用，推动社会治理重心向基层下移。

各位代表，今年是中华人民共和国成立70周年，守底线、防风险、保平安的任务十分艰巨。我们一定要提高政治站位，树牢底线思维，增强忧患意识，认真履职尽责，全力确保社会大局和谐稳定！

三、改革创新、奋发有为，不断提升政府执行力和落实力

自胜者强，自强者胜。新时代、新形势、新任务要求我们必须加快推进学习型、法治型、服务型、务实型、廉洁型政府建设，在推动发展中彰显担当、在落实见效上展示作为。

一是坚持党的领导。坚持以习近平新时代中国特色社会主义思想为指导，深入学习贯彻党的十九大精神，进一步树牢“四个意识”，坚定“四个自信”，做到“两个维护”，坚决在思想上政治上行动上同以习近平同志为核心的党中央保持高度一致，坚决贯彻中央大政方针，创造性地落实省委、省政府和市委的各项决策部署，确保政府工作的正确政治方向。

二是锐意干事创业。政府系统广大干部特别是领导干部要以“改革创新、奋发有为”大讨论为契机，聚焦“六个破除”“六个着力”“六个坚持”，推动学习革命、思想革命、工作革命，激励干部奋发有为、干事创业，凝聚起建设大运城的强大正能量。严格执行末位分析解剖制、重点工作承诺制、重大事项报告制“三项制度”，认真落实“四三”工作机制，强化“五个导向、五个重在”，严格督查问责，不断推动各项工作落地见效。

三是推进依法行政。牢固树立法治理念，自觉运用法治思维和法治方式推动工作。严格执行市人大及其常委会的决议决定，积极配合做好大气污染防治、养犬管理等立法工作，宣传贯彻《运城市农村环境卫生管理办法》，认真办理人大代表建议和政协委员提案，自觉接受市人大的法律监督和市政协的民主监督，主动接受社会各界和新闻媒体舆论监督。建立完善公共法律服务体系，加强法律援助和司法救助。全面推行政务公开，加强行政复议和行政应诉工作。推进市县行政执法体制改革，严格规范公正文明执法，让行政权力始终在阳光下运行。

四是优化营商环境。深化“放管服效”改革，大幅度压缩核准类项目审批评估时限。实施市场准入负面清单制度，全面推行“证照分离”“多证合一”改革，大幅降低准营门槛。进一步完善市县两级政务服务中心功能，全面推行“一窗受理、集成服务”模式，大力发展“互联网+政务服务”，提升行政效能和服务水平。积极落实国家、省各项减税降费政策，坚决制止乱收费、乱罚款行为，切实减轻企业税费负担。崇尚企业家精神，构建“亲”“清”新型政商关系，全力打造“六最”营商环境。

五是加强廉政建设。坚持把纪律和规矩挺在前面，认真履行全面从严治党主体责任，严格遵守党章党规党纪。严格落实中央八项规定精神，坚决反对形式主义、官僚主义。加大审计监督力度，推进公共资金、国有资产、国有资源和领导干部经济责任审计全覆盖。加强财政预算管理，进一步压缩“三公”经费，深入推进政府系统党风廉政建设和反腐败斗争，以为民务实清廉的作风树立政府良好形象。

各位代表，新时代开启新征程，新使命呼唤新作为。让我们更加紧密地团结在以习近平同志为核心的党中央周围，高举习近平新时代中国特色社会主义思想伟大旗帜，在市委的坚强领导下，紧紧依靠全市人民，改革创新、奋发有为，全力推进运城经济社会持续健康发展，为“走进新时代，建设大运城”作出新的更大贡献，以优异成绩向中华人民共和国成立70周年献礼!
